# 3D Embedded Printing of Complex Biological Structures with Supporting Bath of Pluronic F-127

**DOI:** 10.3390/polym15173493

**Published:** 2023-08-22

**Authors:** Tianzhou Hu, Zhengwei Cai, Ruixue Yin, Wenjun Zhang, Chunyan Bao, Linyong Zhu, Honbo Zhang

**Affiliations:** 1 School of Mechanical and Power Engineering, East China University of Science and Technology, Shanghai 200231, China; tristram_hu@outlook.com (T.H.); yinruixue@ecust.edu.cn (R.Y.); 2 Department of Biomedical Engineering, University of Saskatchewan, Saskatoon, SK S7N 5A2, Canada; chris.zhang@usask.ca; 3 School of Chemistry and Molecular Engineering, East China University of Science and Technology, Shanghai 200231, China; caizhengwei@shsmu.edu.cn (Z.C.); linyongzhu@ecust.edu.cn (L.Z.)

**Keywords:** 3D printing, supporting bath, hydrogel, biological structure

## Abstract

Biofabrication is crucial in contemporary tissue engineering. The primary challenge in biofabrication lies in achieving simultaneous replication of both external organ geometries and internal structures. Particularly for organs with high oxygen demand, the incorporation of a vascular network, which is usually intricate, is crucial to enhance tissue viability, which is still a difficulty in current biofabrication technology. In this study, we address this problem by introducing an innovative three-dimensional (3D) printing strategy using a thermo-reversible supporting bath which can be easily removed by decreasing the temperature. This technology is capable of printing hydrated materials with diverse crosslinked mechanisms, encompassing gelatin, hyaluronate, Pluronic F-127, and alginate. Furthermore, the technology can replicate the external geometry of native tissues and organs from computed tomography data. The work also demonstrates the capability to print lines around 10 μm with a nozzle with a diameter of 60 μm due to the extra force exerted by the supporting bath, by which the line size was largely reduced, and this technique can be used to fabricate intricate capillary networks.

## 1. Introduction

In recent decades, 3D printing technology has found widespread applications in the field of tissue engineering [1]. This technology is capable of printing successive layers of materials to fabricate a wide range of structures and complex geometries. Due to its ability to create complex structures from 3D models rapidly and directly, it holds significant potential in surgery and medical equipment [2,3,4,5], as well as scaffolds for tissue engineering. Biodegradable thermoplastic plastics such as polystone and polylactic acid have been widely used as they possess good mechanical properties and allow for the printing of complex and fine structures [6,7,8]. However, these materials usually need to be printed after dissolving with organic solvents or melting at high temperatures, which makes it impossible to print them simultaneously with cells [9]. Hydrogels, on the other hand, possess soft, humid, and good biocompatibility properties and are commonly used as a material in tissue engineering. In 3D printing of tissue scaffolds, the so-called bioink is often used, which encapsulates a large number of cells and hydrogels [10,11,12,13].

Despite their desirable properties, the poor mechanical properties of hydrogels, such as significant shrinkage and difficulty in printing, limit their application as bioinks and make it difficult to build complex models [14,15,16]. To overcome this limitation, adding modifiers to hydrogels is an effective idea [17,18,19]. For instance, Lee et al. added silk fibroin (SF) into gelatin to form the silk fibroin-gelatin (SF-GT) composite hydrogel. Increasing the gelatin content in the composite hydrogel effectively prevented the shrinkage of the composite hydrogel in phosphate buffer solution at 37 °C and enhanced its printability [20]. Liu et al. [21] added chitosan powder to an alginate solution to improve the viscosity of the solution by 1.5–4 times. Increasing the chitosan content also improved the shape fidelity of 3D-printed hydrogels. Wu et al. [22] designed a nanocomposite hydrogel bioink (gelatin–alginate–montmorillonite). This bioink has good printability and mechanical properties based on the thermosensitive properties of gelatin, the advantages of ionic crosslinking of alginate, and the shear thinning and toughening mechanism of nano-MT.

Another idea is using an embedded 3D bioprinting strategy to increase popularity of constructing complex freeform structures. In this strategy, a sacrificial medium is used to support the deposition of bioink in the three-dimensional space before crosslinking. The sacrificial medium rapidly liquefies under yield stress and then solidifies without inducing stress due to its unique shear-thinning and self-healing properties [23]. Lee et al. [24] employed the Freeform Reversible Embedding of Suspended Hydrogels (FRESH) technique, specifically using gelatin microparticles as the sacrificial medium, to successfully print functional ventricles and full-scale human heart models.

Another obstacle in building functional tissues and organs is the difficulty of replicating well-defined vascular networks to facilitate the transport of nutrients and oxygen, maintaining cell viability [25,26]. To address this problem, a sacrificial templating strategy has been applied to construct intricate vascular networks in 3D tissue constructs [27]. In this strategy, a sacrificial ink was first written in the hydrogel matrices; after printing, these matrices were cured and the sacrificial ink was removed, leaving behind a 3D network of interconnected channels [27]. Lewis et al. [28] employed Pluronic F127 bioink to print sacrificial templates with predetermined geometries, followed by casting with cell-laden hydrogel. After printing and crosslinking, the sacrificial template was liquefied, and human umbilical vein endothelial cells (HUVECs) were subsequently seeded to form three-dimensional vascularized tissue, attaining a thickness of approximately 1 cm. Embedded 3D printing techniques also enable the high-fidelity 3D bioprinting of vascular networks. Skylar-Scott et al. [29] developed Sacrificial Writing into Functional Tissue (SWIFT) technology, which uses a suspension medium composed of spheres or organ building blocks (OBBs). The OBB-based suspension medium possesses unique shear-thinning and self-healing properties, enabling sacrificial gel ink printing. Despite its capability to achieve cell densities close to physiological levels in vascularized tissues, the SWIFT technology’s external geometry of printed tissues is significantly constrained by the container, thus limiting the faithful replication of native organ geometries like ventricles. To simultaneously replicate the external geometry and complex internal structures of functional tissues, Fang et al. [30] developed the Sequential Printing via Inkjet-deposited Templates (SPIRIT) technique. By using several independent printing stages, the external shape and internal structure of the tissue are formed separately, resulting in a ventricle model with a perfusable vascular network. Although this technology achieves the printing of organ-specific tissues, it heavily relies on a cell-laden hydrogel or bio-material that can serve as both the embedded bioink and the suspension medium for bioprinting. This introduces some limitations in the selection of materials for bioinks. Additionally, with the SPIRIT technique, only 500 μm-sized vessels were printed, and it is still challenging to print capillaries at the scale of tens of micrometers or even micrometers.

In this study, we present a PF-127 supporting bath printing technology for fabricating complex biological models using hydrogels. Utilizing a variety of bioinks with distinct viscosity and crosslinking mechanisms, we effectively prevent the deformation of soft structures during the printing process, thanks to the support provided by the PF-127 bath. This technology enables the successful printing of microlines with a width of 10 μm, representing a significant breakthrough in overcoming the current size limitation of DIW technology. Additionally, it plays a crucial role in fabricating intricate capillary networks. Furthermore, we ensure biocompatibility throughout the entire printing process by utilizing aqueous environments that are tightly controlled for pH, ionic strength, temperature, and sterility. Our study objectives encompassed the utilization of diverse bioinks, prevention of soft structure deformation, the realization of microline printing at the 10-micrometer scale, and ensuring biocompatibility through meticulous control of environmental parameters.

## 2. Material and Methods

### 2.1. Bioink Preparation

#### 2.1.1. GelMA Bioink

To prepare the GelMA bioink, 1 g of gelatin (type A, purchased from Sigma-Aldrich, St. Louis, MO, USA) was dissolved in 10 mL of DPBS solution at 50 °C. Then, 2 mL of Methacrylic anhydride (MA, purchased from Adamas beta, Shanghai, China) was added to the solution to obtain a mixed solution with a final concentration of 20% (*v*/*v*). The mixed solution was stirred using magnetic force in a 50 °C environment for 3 h. Subsequently, the solution was diluted with DPBS (50 °C) to a final volume of 60 mL. The solution was then centrifuged at 8000 rpm for 10 min to remove unreacted MA and other by-products. The centrifuged solution was poured into a dialysis bag (MWCO 14000 Spectrum^®^, Solarbio, Beijing, China) and dialyzed in deionized water at 37 °C for 5 days, during which the deionized water was replaced every 12 h. After dialysis, the GelMA solution was freeze-dried at −60 °C for 48 h, and the resulting freeze-dried GelMA could be stored at 4 °C.

To make the GelMA bioink, 100 mg of the freeze-dried GelMA was dissolved in 1.9 mL of deionized water. Then, 3 mg of LAP (from State Key Laboratory of Bioreactor Engineering, East China University of Science and Technology) was added as a photo-initiator to obtain the GelMA bioink with a concentration of 5%. The resulting bioink can be crosslinked using 365 nm ultraviolet (UV) light.

#### 2.1.2. HAMA Bioink

To prepare HAMA bioink, 1 g of sodium hyaluronate (HA, 300,000, purchased from Blomade Freda Biopharm, Shandong, China) was dissolved in 100 mL of deionized water and maintained at 4 °C for subsequent reactions. An amount of 8 mL of MA was added to the solution, and the pH was adjusted to 8 with NaOH. The solution was then allowed to react for 12 h before being transferred to a dialysis bag and dialyzed with deionized water for 24 h, with the dialysis water being replaced every 8 h. The solution was then centrifuged at 7000 rpm for 10 min to remove unreacted precipitation, and the supernatant was dialyzed again for 3 days. After dialysis, the solution was frozen at −80 °C for 3 h and then freeze-dried in a freeze dryer for 2 days to obtain HAMA. To prepare HAMA bioink, 60 mg of HAMA was dissolved in 1.94 mL of deionized water, and 3 mg of LAP was added as a photo-initiator to obtain a HAMA bioink with a concentration of 3%, which can be crosslinked by 365 nm UV light.

#### 2.1.3. PF-127DA Bioink

To prepare PF-127DA bioink, 400 mg of PF-127DA (from State Key Laboratory of Bioreactor Engineering, East China University of Science and Technology) was dissolved in 1.6 mL of deionized water, and 3 mg of LAP was added as a photo-initiator to obtain a PF-127DA solution with a concentration of 3%, which can be crosslinked by 365 nm UV light.

#### 2.1.4. Alginate Bioink

To prepare Alginate bioink, 50 mg of Alginate was dissolved in 1.95 mL of deionized water to obtain Alginate bioink with a concentration of 2.5%, which can be crosslinked by Ca^2+^ ions in the supporting bath.

### 2.2. PF-127 Supporting Bath Preparation

#### 2.2.1. General PF-127 Supporting Bath

An amount of 6 g of Pluronic F-127 (PF-127, purchased from Sigma-Aldrich) powder and 30 mL of deionized water (4 °C) was mixed with a vortex mixer at 3000 rpm for 30 min, then store in a refrigerator at 4 °C for 2 h and mix again for 10 min. Repeat this operation 5 times to obtain a final concentration of 20% (*w*/*v*) PF-127 supporting bath. Prepare PF-127 supporting baths with mass volume ratios of 25%, 30%, 35% and 40% according to the same procedure, seal and store in a −4 °C refrigerator. Before use, remove the supporting bath from −4 °C, place it in at 4 °C to thaw for 12 h, and then mix it on a vortex mixer for 10 min.

#### 2.2.2. PF-127 Supporting Bath with Ca^2+^ Ions

An amount of 60.5 mg of CaCl_2_ powder was dissolved in 50 mL of deionized water to make 11 mM CaCl_2_ solution and stored at 4 °C. 7.5 g of PF-127 powder and 30 mL of CaCl_2_ solution (4 °C) was mixed according to the above steps to obtain a final concentration of 25% (*w*/*v*) PF-127 supporting bath with Ca^2+^ ions.

### 2.3. PF-127 Supporting Bath Performance Evaluation

#### 2.3.1. Rheology Test

The rheological properties of PF-127 supporting bath were evaluated using a HAAKE Mar III rheometer (OmniCure Series 2000, Thermo Fisher Scientific, Waltham, MA, USA) at concentrations of 20%, 25%, 30%, 35%, and 40%. Two parameters, G′-T and G″-T, were measured to characterize the storage modulus (G′, in Pa) and the loss modulus (G″, in Pa) as a function of temperature (T, in °C). A 20 mm P20 TiL disc was used as the parallel plate, and the test conditions were set as ƒ = 1 Hz and Γ (rotor deflection) = 10%. Each set of data was tested in triplicate to ensure the reproducibility of the results.

#### 2.3.2. Scratch Recovery Test

To evaluate the scratch recovery effect of the PF-127 supporting bath at different concentrations, a needle with an outer diameter of 1260 μm was used to scratch the surface of the gel at a distance of 7 mm from the top. Tests were performed in 20%, 25%, 30%, 35%, and 40% PF-127 supporting bath, which was stabilized for 2 h at a temperature of 26 ± 1 °C to reach a soft gel state. The depth of the scratch mark was measured to evaluate the scratch recovery effect.

### 2.4. PF-127 Support Bath Printing Effect on Printing Accuracy

The printing accuracy of the 3D bioprinter (3D Bioplotter; EnvisionTec, Gladbeck, Germany) was assessed using PF-127DA bioink, different printing needles with varying inner diameters, and PF-127 supporting baths with different concentrations. To evaluate the single-line accuracy of printing, needles with inner diameters of 60 μm, 90 μm, 160 μm, and 210 μm were used to print in a PF-127 supporting bath with a concentration of 25%. In a separate test, a needle with an inner diameter of 60 μm was used to print in PF-127 supporting baths with concentrations of 20%, 25%, 30%, 35%, and 40%. The influence of the PF-127 supporting bath on printing accuracy was analyzed.

### 2.5. PF-127 Support Bath Printing Compatibility Test

#### 2.5.1. Compatibility of Different Bioinks

All printing was performed using a PF-127 supporting bath with a concentration of 25%. GelMa, HAMA, and PF-127DA bioinks were cross-linked using 365 nm UV light after printing in the PF-127 supporting bath. Alginate bioink was printed in a supporting bath with Ca^2+^ ions and directly crosslinked by Ca^2+^ ions.

#### 2.5.2. Biocompatibility

Human dermal fibroblast (HDF, Shanghai Jiaotong University Ninth People’s Hospital, Shanghai, China) cells were cultured in DMEM high glucose medium (Hyclone Company, Logan, UT, USA) with 10% fetal bovine serum (FBS) and 1% penicillin-streptomycin in a commercial incubator (160i; Thermo Fisher Scientific) at 37 °C with 5% CO_2_.

PF-127DA bioink was used to print complex structures using a PF-127 supporting bath of 25%. After printing, the scaffold was cross-linked using 365 nm UV light. The supporting bath was cooled down to 4 °C to make the PF-127 liquid remove the scaffold and wash off the remaining PF-127 on the surface of the scaffold. The scaffold was then immersed in 0.012 mg/mL collagen (rat tail, Corning, Corning, NY, USA) for 12 h to allow the collagen to completely infiltrate the internal pores of the scaffold. The collagen-coated scaffolds were used for in vitro experiments.

Thereafter, 2 × 104 HDF cells suspended in 50 μL DMEM high glucose medium were seeded on PF-127DA scaffolds to form PF-127DA-HDF constructs. Nonadherent cells were washed away one day after seeding, and the scaffolds were transferred to a blank well plate. The scaffolds were cultured in DMEM high glucose medium with 10% FBS and 1% penicillin-streptomycin in an incubator at 37 °C with 5% CO_2_. Cell Counting Kit-8 (Dojindo, China) was used to test cell proliferation at 1, 4, and 7 days.

### 2.6. Statistical Analysis

The data were expressed as mean ± standard deviation (SD). The statistical analysis was performed using the one-way ANOVA (analysis of variance) test to determine significant differences. A *p*-value < 0.05 was considered statistically significant.

## 3. Result and Discussion

The use of PF-127 as a supporting bath has demonstrated its potential as a highly adaptable and cost-effective bio-AM platform. The key advantage of this technology is that the printed hydrogel is deposited and embedded in the PF-127 support bath, which maintains the expected structure during the printing process and significantly improves the printing fidelity. The PF-127 hydrogel system is thermosensitive, existing as a liquid at low temperature and becoming a semi-solid gel at higher temperatures. As a result, the hydrogel that is squeezed out of the nozzle and deposited in the bath stays in place, allowing soft materials that slump when printed in the air to remain in their intended 3D geometry in the support bath.

### 3.1. Performance of PF-127 Supporting Bath

**Figure 1:**
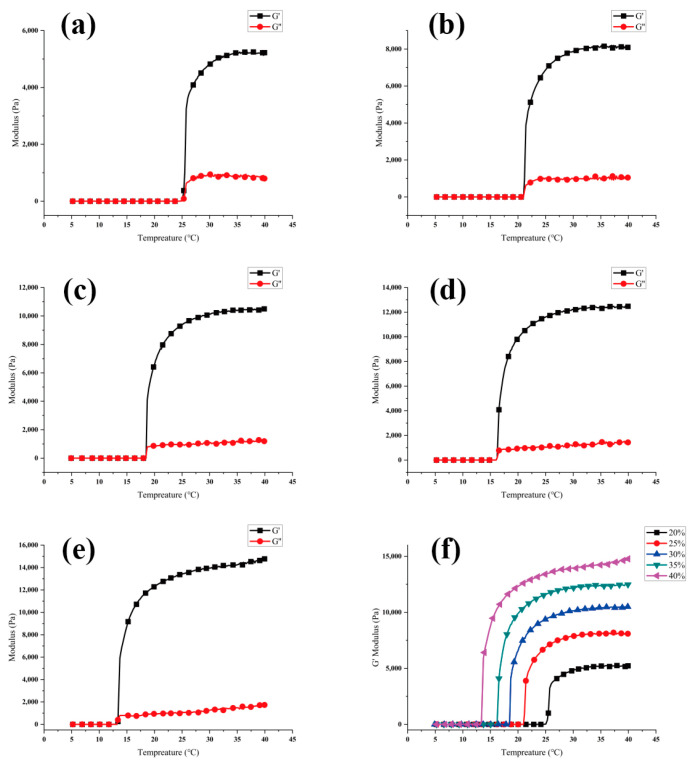
The relationship between G′-T and G″-T of PF-127: (**a**) 20% PF-127; (**b**) 25% PF-127; (**c**) 30% PF-127; (**d**) 35% PF-127; (**e**) 40% PF-127; (**f**) G′ comparison at 20%, 25%, 30%, 35% and 40% PF-127 concentration.

**Figure 2:**
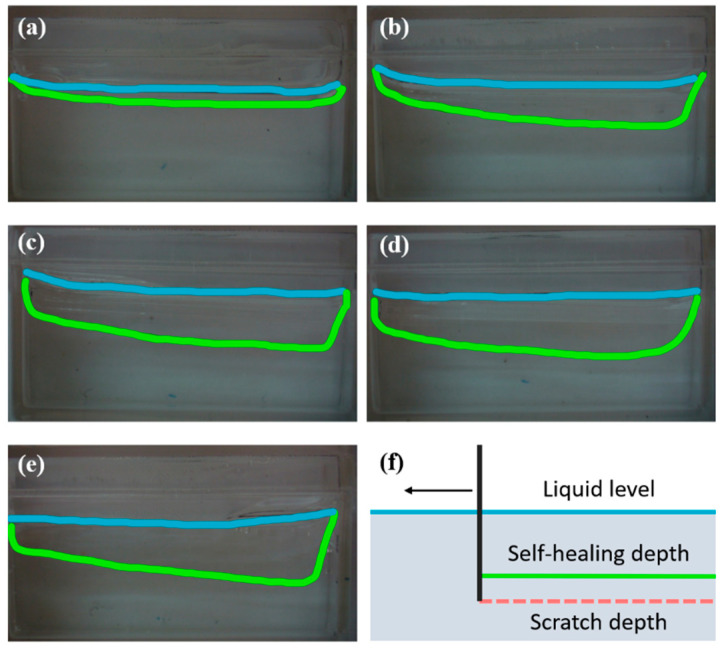
Scratch depth result in PF-127 support bath: (**a**) 20% PF-127; (**b**) 25% PF-127; (**c**) 30% PF-127; (**d**) 35% PF-127; (**e**) 40% PF-127; (**f**) schematic diagram of scratch test. (The blue lines illustrate the liquid level and the green lines illustrate the self-healing depth after the scratch).

During the printing process, the support bath must have sufficient storage modulus to act as a support for the bioink while quickly recovering scratches for the next layer of support. Rheological tests have shown that increasing the solid content of PF-127 improves its support performance by increasing its G′ while decreasing its critical phase transition temperature from 25 °C to 13 °C (as shown in Figure 1). However, under the condition of 25 ± 1 °C, increasing the solid content of the support bath leads to deeper scratch depth and worse healing effects (as shown in Figure 2).

### 3.2. PF-127 Support Bath Printing of Microline

Hydrogels experience extrusion swelling during the extrusion printing process in air. Upon extrusion from the needle, the diameter of the extrudate can be significantly larger than the size of the needle port, sometimes swelling more than twice its original size. This swelling is caused by the hydrogel’s elastic memory ability. When the colloid enters the needle, it undergoes strong stretching and shearing deformation. The stretching deformation is elastic, and only a portion of these deformations is relaxed at the needle’s exit. The remaining part causes elastic recovery of the hydrogel after extrusion, leading to extrusion swelling.

**Figure 3:**
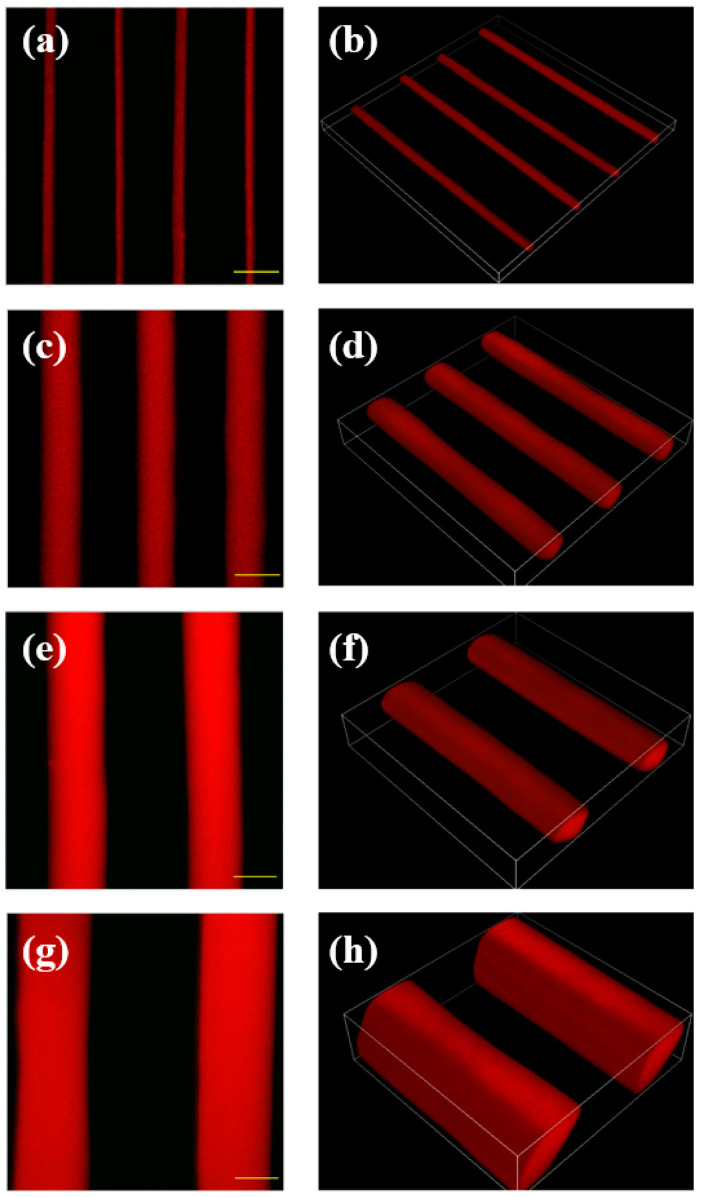
Different size needle’s printing effect in 25% concentration of PF-127 support bath: (**a**,**b**) 60 μm; (**c**,**d**) 90 μm; (**e**,**f**) 160 μm; (**g**,**h**) 210 μm (scale bars = 100 μm).

**Figure 4:**
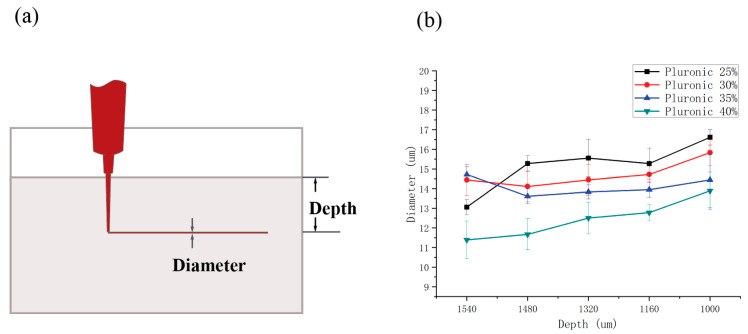
PF-127 Support Bath Printing of microline: (**a**) schematic illustration of the microline print in supporting bath. (**b**) Needle prints at 60 μm line diameter in different support bath environments.

The PF-127 supporting bath has a beneficial effect on the printed bioink by reducing extrusion swelling. When printed with needles of different inner diameters (60 μm, 90 μm, 160 μm, and 210 μm), clear line structures can be obtained, and the printed material line diameters are smaller than the inner diameter of the needles (Figure 3). Furthermore, the higher the concentration of the PF-127 supporting bath or the farther the printing position from the surface of the supporting bath, the more prominent the extrusion effect of the supporting bath on the printing lines, leading to higher accuracy of single-line printing. In fact, the accuracy of single-line printing in the PF-127 supporting bath can reach up to 10 μm, far exceeding the printing accuracy without using this technology (Figure 4). 

The PF-127 supporting bath applies additional pressure to the hydrogel printed line, leading to the compaction of its internal porous structure. The initially expanded pores will contract, resulting in an overall reduction in the hydrogel’s volume. As a consequence, the microline’s diameter becomes smaller than the nozzle diameter. As the concentration of the supporting bath or the printing position depth increases, the microline’s diameter experiences more significant shrinkage (as shown in Figure 4b).

### 3.3. Compatibility of Different Bioinks

**Figure 5:**
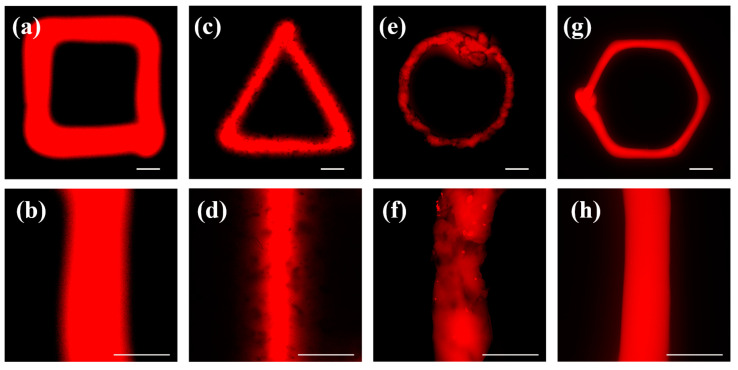
Printing of different materials in 25 wt% of PF-127 support bath: (**a**,**b**) HAMA; (**c**,**d**) Alginate; (**e**,**f**) GelMA; (**g**,**h**) PF-127DA (scale bars = 200 μm).

**Figure 6:**
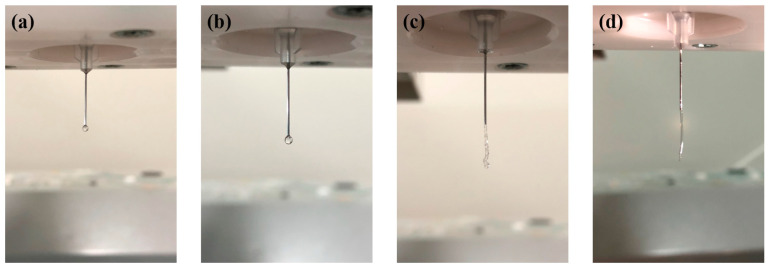
Extrusion of different materials from the printing nozzle: (**a**) HAMA; (**b**) alginate; (**c**) GelMA; (**d**) PF-127DA.

**Figure 7:**
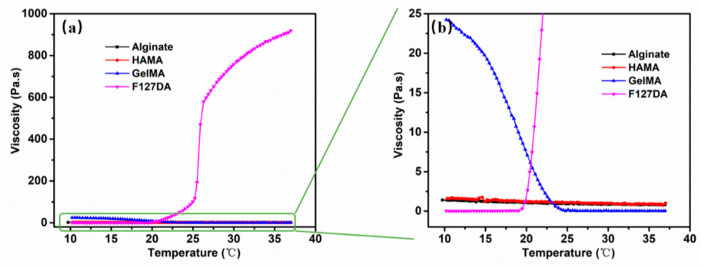
The relationship between viscosity and temperature of different printing material: (**a**) The relationship between Viscosity–T of Alginate, HAMA, GelMA and PF-127DA; (**b**) detailed view.

The PF-127 supporting bath printing technology is compatible with various bioinks that have different viscosities and curing methods (as shown in Figure 5). The printing effect depends on the bioink’s characteristics. PF-127DA produces thin, well-defined lines with uniform thickness. This is due to the material’s significant temperature sensitivity. At a controlled temperature of 37 °C, the PF-127DA material extrudes in a filamentous shape, as depicted in Figure 6. This bioink remains in its extrusion state while in the supporting bath at a controlled temperature of 25 °C (as shown in Figure 7), making it easy to achieve excellent printing resolution.

The lines printed with HAMA bioink are significantly thicker than those printed with other bioinks. This is because the viscosity of the HAMA bioink cannot be adjusted by temperature during the printing process. The low viscosity bioink diffuses into the needle’s scratch before the supporting bath can recover, leading to a printing width that corresponds to the needle’s outer diameter rather than its inner diameter.

The line boundaries printed with alginate bioink are less defined and have uneven thickness. This is because alginate’s crosslinking mechanism differs from that of the other three bioinks. PF-127DA, HAMA, and GelMa are crosslinked by UV light after printing, whereas sodium alginate reacts directly with Ca^2+^ ions in the supporting bath while printing, resulting in less-sharp boundaries in the printed samples that are more affected by the printing process.

### 3.4. Biocompatibility of PF-127 Supporting Bath

**Figure 8:**
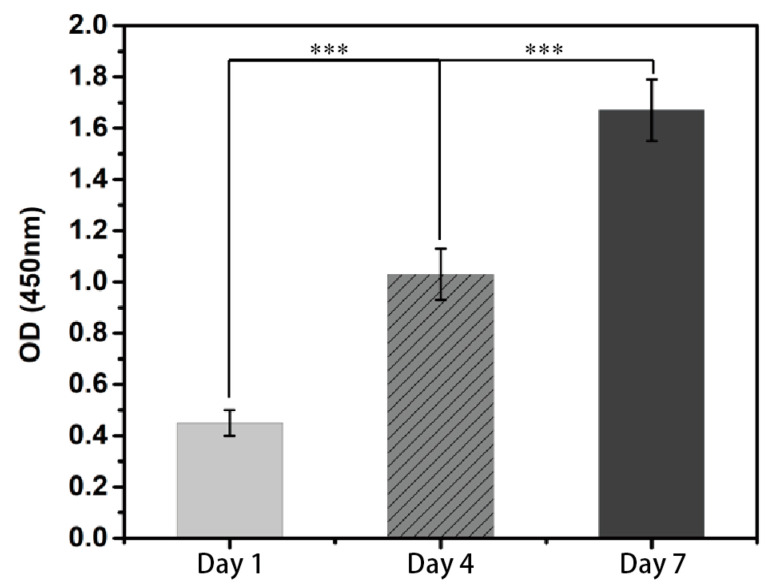
Proliferation of HDF cells during 7 days of culture (*** means *p* < 0.01).

The Cell Counting Kit-8 experiment conducted on the PF-127DA-HDF constructs demonstrated that the cells on the scaffold exhibited a robust proliferation state with the passage of culture time (as illustrated in Figure 8). The scaffolds produced by the PF-127 supporting bath technology exhibited no cytotoxicity and displayed excellent biocompatibility.

### 3.5. 3D Printing of Complex Biological Structures

**Figure 9:**
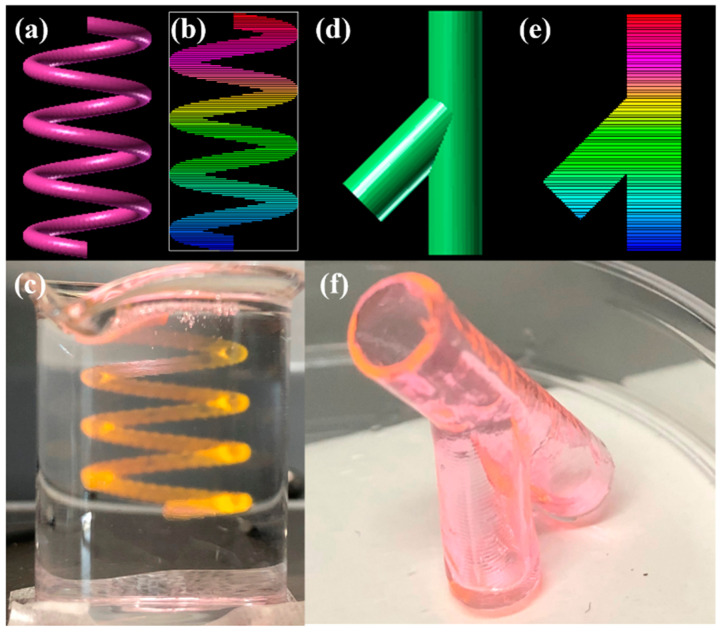
Spring and Y-tube model printing effect: (**a**) Model graphic of spring model; (**b**) Slice graphic of spring model; (**c**) physical graphic of spring model; (**d**) model graphic of Y-tube model; (**e**) slice graphic of Y-tube model; (**f**) physical graphic of Y-tube model.

**Figure 10:**
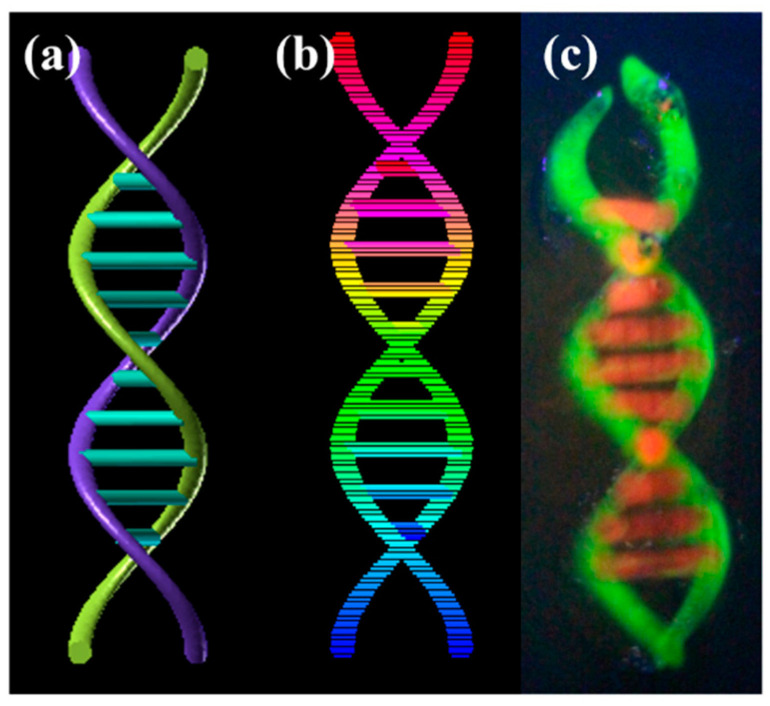
DNA model printing effect: (**a**) Model graphic of DNA model; (**b**) slice graphic of DNA model; (**c**) physical graphic of DNA model.

**Figure 11:**
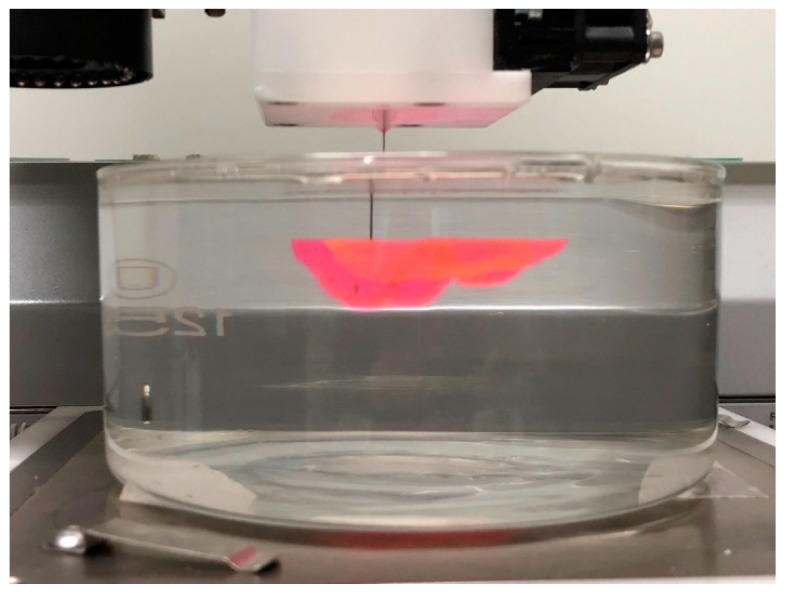
Full ear model printing process in the support bath.

**Figure 12:**
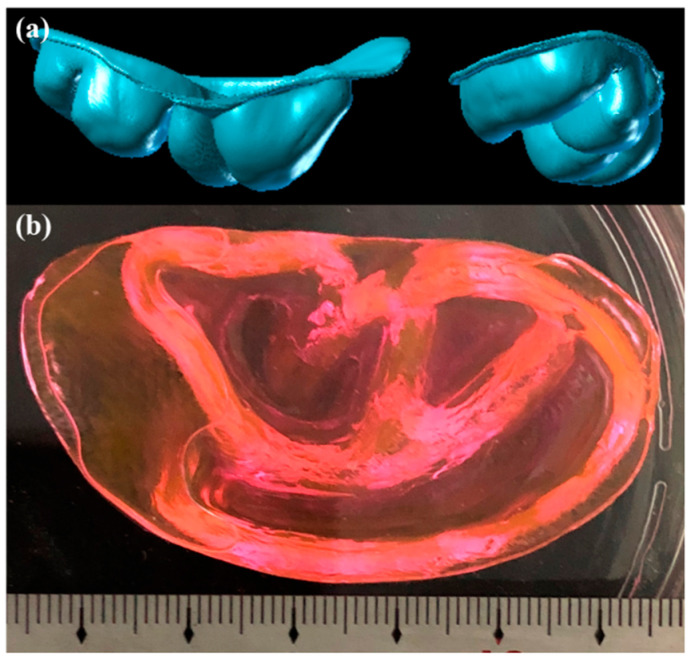
Full ear model printing result: (**a**) Model graphic of full ear model; (**b**) physical graphic of full ear model.

Subsequently, the supporting bath printing technology was employed to 3D print complex biological structures based on medical imaging data in order to showcase its capacity to fabricate intricate geometries. Initially, we employed the PF-127 support bath printing technology to print springs and DNA helical structures that are challenging to realize in a conventional printing environment (as depicted in Figure 9 and Figure 10). Subsequently, we 3D printed a human ear using PF-127DA bioink, and the printed ear exhibited a high-fidelity resemblance to the actual human ear (as shown in Figure 11 and Figure 12). Furthermore, to demonstrate the ability of the supporting bath technology to print a hollow structure, we designed and printed a simple bifurcated tube (height of 30 mm, O.D. of 4.5 mm, I.D. of 4.0 mm) using CAD. Across all these tests, the supporting bath printing technology demonstrated excellent forming capabilities.

## 4. Conclusions and Future Aspects

In this paper, we employed HAMA, GelMA, PF-127DA, and alginate bioinks to demonstrate the versatility of embedded 3D printing technology using a supporting bath coupled with photopolymerization or chemical crosslinking mechanisms. The supporting bath technology successfully replicates the external geometry of native organs, as evidenced by the successful printing of a whole ear model. Furthermore, using the supporting bath, we successfully printed microlines at a scale of 10 μm. The additional pressure provided by the supporting bath results in the diameter of the printed bioink being significantly smaller than the nozzle diameter. The HDF cell proliferation test on the scaffold printed using the supporting bath technology showed no cytotoxicity, indicating excellent biocompatibility.

Achieving simultaneous replication of both external organ geometry and intricate internal structures of native tissues is a goal that necessitates further research and development for its full realization. However, many companies and academic laboratories are actively working towards this goal [11,13,26,31]. Supporting bath bioprinting technology offers robust compatibility with multiple bioinks, enabling the printing of native tissue’s external organ geometry and facilitating the replication of intricate capillary networks. This technology has the potential to expand bioprinting into a wide range of academic and commercial laboratory settings, accelerating important breakthroughs in tissue engineering for applications ranging from drug testing to regenerative therapies. Further research is needed to optimize the printing process, develop new bioinks, and improve the biocompatibility of printed constructs. With continued innovation and advancement, supporting bath printing technology has the potential to revolutionize tissue engineering and bring us one step closer to the ultimate goal of bioprinting functional organs for human transplantation.

## Data Availability

The data presented in this study are available on request from the corresponding authors.
